# High Density Static Charges Governed Surface Activation for Long-Range Motion and Subsequent Growth of Au Nanocrystals

**DOI:** 10.3390/nano9030328

**Published:** 2019-03-01

**Authors:** Guoxin Chen, Changjin Guo, Yao Cheng, Huanming Lu, Junfeng Cui, Wanbiao Hu, Rongrong Jiang, Nan Jiang

**Affiliations:** 1 Key Laboratory of Marine Materials and Related Technologies, Zhejiang Key Laboratory of Marine Materials and Protective Technologies, Ningbo Institute of Materials Technology & Engineering, Chinese Academy of Sciences, Ningbo 315201, China; gxchen@nimte.ac.cn (G.C.); hmlu@nimte.ac.cn (H.L.); cuijunfeng@nimte.ac.cn (J.C.); jiangrr@nimte.ac.cn (R.J.); 2 School of Materials Science and Engineering, Yunnan University, Kunming 650091, China; guochangjin@mail.ynu.edu.cn; 3 Fujian Institute of Research on the Structure of Matter, Chinese Academy of Sciences, Fuzhou 350002, China; chengyao@fjirsm.ac.cn; 4 Center of Materials Science and Optoelectronics Engineering, University of Chinese Academy of Sciences, 19A Yuquan Rd., Shijingshan District, Beijing 100049, China

**Keywords:** Au nanocrystals, charging, surface activation, long-range motion, in-situ TEM

## Abstract

How a heavily charged metal nanocrystal, and further a dual-nanocrystals system behavior with continuous electron charging? This refers to the electric dynamics in charged particles as well as the crystal growth for real metal particles, but it is still opening in experimental observations and interpretations. To this end, we performed an in-situ electron-beam irradiation study using transmission electron microscopy (TEM) on the Au nanocrystals that freely stand on the nitride boron nanotube (BNNT). Au nanocrystalline particles with sizes of 2–4 nm were prepared by a well-controlled sputtering method to stand on the BNNT surface without chemical bonding interactions. Au nanoparticles presented the surface atomic disorder, diffusion phenomena with continuous electron-beam irradiation, and further, the long-range motion that contains mainly the three stages: charging, activation, and adjacence, which are followed by final crystal growth. Firstly, the growth process undergoes the lattice diffusion and subsequently the surface-dominated diffusion mechanism. These abnormal phenomena and observations, which are fundamentally distinct from classic cases and previous reports, are mainly due to the overcharging of Au nanoparticle that produces a surface activation state in terms of high-energy plasma. This work therefore brings about new observations for both a single and dual-nanocrystals system, as well as new insights in understanding the resulting dynamics behaviors.

## 1. Introduction

Classical electromagnetics theory clearly tells all of the electrical-related behaviors e.g., charge spatial/time distributions, potential fields, interactions, movements, etc. for a charged particle or system in a magnetoelectric field. These are undoubtfully correct, as long-term theory is established mainly based on the relatively ideal particles or systems, while the cases become quite complicated for the real particles and systems, because, especially for the nanoscale particles, the shape stability, atomic configurations, surface reconstruction, thermal effects, electron flux fluctuations, over-charged phenomena, etc. should be taken into account, which makes it unsurprising if some abnormal or even quite different phenomena happen [1]. For instance, Gutiérrez et al. [2] observed the nanoscale particle (NP) fusion in alkanethiol passivated Au crystals where the interactions between thiol and NP surface led to destabilization in surface Au atoms, yielding surface distortions to cause loosely-held or ad-atoms. The involved ad-atoms and coalescence behaviors, which are thought to be due to the attraction potential of adjacent NPs, are obviously different to the classic/ideal cases. It is thus highly necessary to uncover and further understand the new phenomena and natures, which are directly related to the new explorations and design in the crystal growth, electrical, magnetic optical, and catalytic properties [3].

One sole-element metal particle system is the simplest but closely connected case for addressing aforementioned questions in this regard. The electrical and dynamic behaviors under eternally charging conditions of metallic atoms and clusters on the carbon and/or graphene were recently extensively studied [4,5,6,7,8,9]. Motion behaviors of single metallic atoms and clusters on a carbon support were observed [10], and more recently, Batson et al. [11] also presented the similar phenomenon. To gain better insights, atom dynamics, surface sputtering in Pt clusters, and elongation behaviors of Au clusters under parallel electron beam irradiations were studied by Karbasi and Han et al., respectively [12], but systematic investigations and interpretations on these abnormal phenomena, in order to reach a comprehensive model at a fundamentally atomic level, are still lacking.

Among these differently involved metal-particle systems, Gold (Au) is considered to be an appropriate candidate because of its high stability and chemical inertia, while spatial/time monitoring and characterizations of nanoscale Au particles would be subject to in-situ transmission electron microscopy (TEM), which allows the real-time structural characteristics of shape, size, surface, atom, etc., and, more importantly, directly provides electron-beam and charging conditions [13]. A study by Yuk et al. [5] on the photo-assisted fusion of Au NPs on graphene revealed two distinct coalescence pathways, which involved the interface elimination between particles, producing defect-free NP fusion. Electron-beam driven structural dynamics for metallic nanoparticles was also observed, for instance, by Smith et al., who reported structural rearrangements of Au nanoparticles [14]. Whereas, these new and singular phenomena are quite fascinating, their behaviors are dependent of, on the one hand, the particles’ intrinsic natures e.g., heat and charge conductance, geometry and orientation, atomic bond energies, and local chemical environment, etc., and on the other, the electron energy and dose of the electron beams and perhaps the substrates used [15,16]. This often gives a complex interplay of different motion and damage mechanisms, which are sometimes inconsistent or even controversial in understanding such sorts of behaviors and phenomena.

It is especially noted that the most studies above for the loading substrates are mainly performed on carbon/graphene. However, these single-element atoms are often bonded to the carbon surface that is strong enough to remain in one spot for a long period under the electron beams [11]. As a consequence, such experimental strategies may suffer from a contact interface existing between metal nanoparticles and the substrates, resulting in the observations not being authentic or intrinsic. To avoid these shortcomings, a more appropriate substrate and better design could be based on boron nitride nanotubes (BNNTs), which, when comparing to carbon-based substrates, exhibit higher chemical stability against oxidation, higher thermal conductivity and mechanical stability [17,18]; furthermore, BNNTs display larger resistance and uniform electronic bands that are not influenced by the tube chirality and diameters, making them ideal as supports for a broad range of nanomaterials and properties [19,20]. 

In this work, we explored an in-situ spatial/time TEM electron-beam charging strategy on Au nanocrystals that freely stand on and without any bonding to BNNT support in order to investigate the dynamic behaviors of a charged real particle and particle system to establish the physical pictures and the involved mechanism under electric fields. To our best knowledge, it is the first time to observe the Au nanoparticles (2–4 nm) that experienced anomalous diffusive motion and subsequent crystal growth with surprisingly high rates, through a surface activation mechanism to fulfill long sliding movement. The high density static charging induced surface activation e.g., atomic diffuse disorder and plasma, which were caused by the charger transfer between insulator BNNTs and Au nanocrystals under electron beam, is responsible for these behaviors and phenomena.

## 2. Materials and Methods

### 2.1. Synthesis of AuNPs@BNNTs

High-quality BNNTs with uniform morphology in tube length, diameters, and number of layers were purchased from Nanjing XFNANO Materials Tech Co., Ltd. (Nanjing, China) 10 mg BNNTs powders were well dispersed into 10 mL chloroform ultrasonically with a duration of 15 min to obtain suspension. Subsequently, this suspension with BNNTs was casted on clean glass slide and then dried after 30 min to completely evaporate the chloroform. Afterwards, the physical sputtering method was employed to deposit Au nanoparticles on the BNNTs surface. The sputter current and time were set to 10 mA and 10 s, respectively, to control the Au particle size well. The advantage of this method would be to not allow the formation of strong chemical bonding between Au nanoparticles and BNNTs substrate, unlike those chemical methods that often cause strong chemical bonding that induces the unexpected interactions, making the in-situ experiments not authentic. 

### 2.2. Specimen Preparation

The AuNPs@BNNTs powders were collected and dispersed in acetone and then drop-casted on 300 mesh ultrathin carbon film on holey carbon support copper grids for TEM measurements. The sample was directly transferred on the Si substrate for XPS measurements (Shimadzu AXIS ULTRA DLD, Manchester, UK).

### 2.3. TEM Imaging and Data Analysis

Field emission transmission electron microscopy (ThermoFisher Talos F200X, Brno, Czech Republic) with a super bright electron gun was used to carry out the in-situ experiments. For TEM observations, the operation voltage was set at 200 keV, the magnification was set to 630 kX, and the electron-beam intensity was set to 10^5^ A/m^2^, unless otherwise stated. Liquid nitrogen was used externally to cool a cold finger in order to reduce sample contaminations. The experiment videos were recorded at a rate of 10 fps by CMOS camera. When a region of interest was found, we changed the spot size and intensity in order to set the target electron-beam intensity and then start recording the video under 630 kx. It should be noted that the electron beam was used to illuminate the samples, only at the moment of taking micrographs during the experiment. This helps to avoid the electron beam effect as much as possible. The data analysis used Velox software (Thermal Fisher Company, Waltham, MA, USA), which can help to extract image from video and make FFT of interest area.

## 3. Results

**Figure 1:**
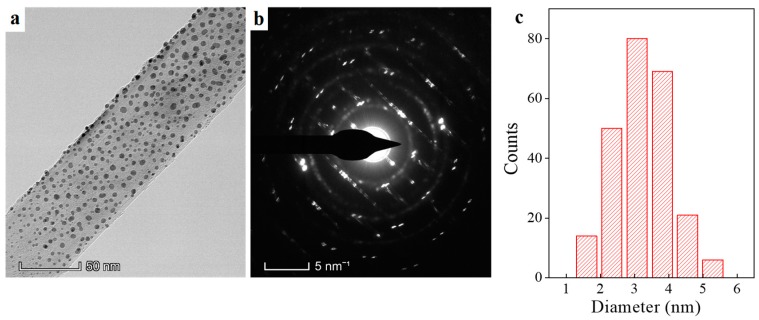
Transmission electron microscopy (TEM) characterizations of the AuNPs@boron nitride nanotubes (AuNPs@BNNTs). (**a**) Large-scale TEM observation of AuNPs@BNNTs; (**b**) Electron diffraction pattern corresponding to Figure 1a selected area; and, (**c**) Particle size distribution of Au NPs on BNNTs, which is based on the size measurements of 240 Au nanoparticles.

Figure 1 shows the overall characteristics of the as-prepared AuNPs@BNNTs. It can be seen that Au NPs are uniformly distributed on the surface of the BNNTs substrate. These Au NPs have sharp shapes, which are indicative of good crystallinity. Correspondingly, the selected area electron diffraction (SAED) pattern (Figure 1b clearly gives diffraction rings of (200), (111), (220), and (222) that are indexed to face-center cubic structure Au NPs, while the elongated diffraction dots correspond to hexagonal BNNTs. Furthermore, these Au NPs exhibit very narrow particle size distributions of dominated around 2–4 nm, which have compatible surface energy. To conclude, the present AuNPs@BNNTs system has been designed in high quality with excluded multiple uncertainties to improve the reliability.

**Figure 2:**
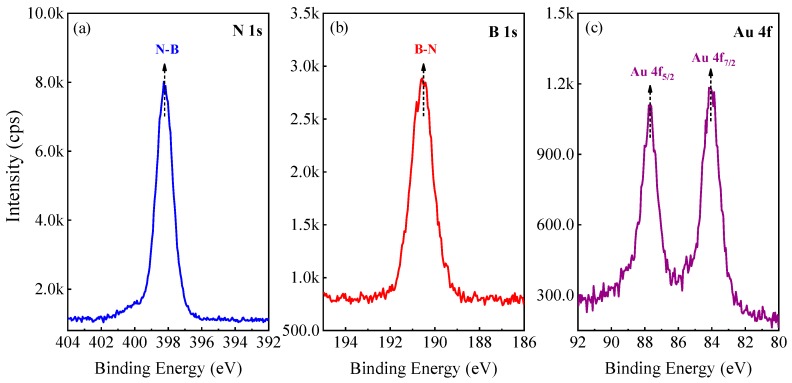
X-ray photoelectronic spectroscopy (XPS) spectra of N, B, and Au in AuNPs@BNNTs.

The states of the chemical bonding between Au NPs and BNNTs were examined by X-ray photoelectronic spectroscopy (XPS). Figure 2a shows the B1s peaks for the Au@BNNTs, which is quite symmetric and centered (after charging effect correction with C1s) at approximately 191.5 eV, as consistent with previous photoemission investigations [21]. Other possible components below the main peak, e.g., from the B-O and B-C bonds, are not resolved in our spectra. Similarly, the N1s peak of BNNTs (Figure 2b) is also symmetric with a binding energy that is located at 398.8 eV, as expected for N-B bonds in pure hexagonal NB. The Au 4f_5/2_ and 4f_7/2_ profiles with their energy of 87.7 eV and 84.1 eV, both in the peak shape and location, are identical to the pure Au NPs [22]. These XPS results suggest that there are no interactions (or negligible) e.g., chemical bonding between Au NPs and BNNTs, unlike the carbon/graphene-metal systems that may impose great effects upon the electric and/or motion behaviors under the charging conditions addressed later.

**Figure 3:**
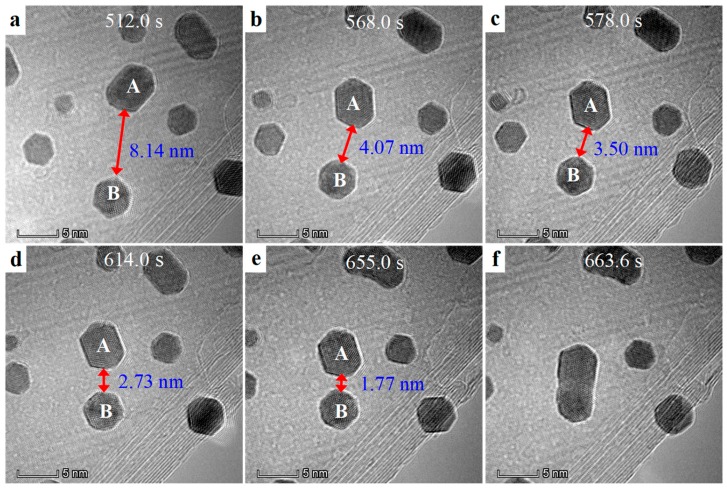
**A typical time series of TEM image extract from the video (Supplementary Materials-1).** Long-range motion and subsequent growth process of Au NPs on BNNTs at a top view under TEM e-beam irradiation. A sequence of images collected at different irradiation time for two selected Au NPs (highlighted by A and B) ware demonstrated. These two Au NPs experienced firstly rotating, then moving, and finally growth under e-beam irradiation.

Figure 3 demonstrates the dynamic behaviors of the Au NPs under TEM e-beam irradiation. To clearly depict the experimental phenomena, we mainly focused on two Au NPs (highlighted by A and B in Figure 3) at a top view (corresponding video was show in Figure S1). These two Au NPs was separated by a long distance of over eight nanometers (Figure 3a). Under the e-beam irradiation, these two Au NPs behaved no differently at the beginning in a long period of around 512 s, which is perhaps due to the particles charging, as will be addressed later. While, after 512 s, both the Au NP started to firstly rotate, and then gradually move towards each other with the continuous e-beam irradiation (Figure 3b). It is interesting that, finally, these two Au NPs moved to touch with each other and very quickly merge (at around 0.1 s level) into one particle. The most striking scenery thereby is that the Au NPs clearly readily migrated over 8.3 nanometers in distance, much larger than the coalesced critical edge-to-edge distance in other reports [8].

**Figure 4:**
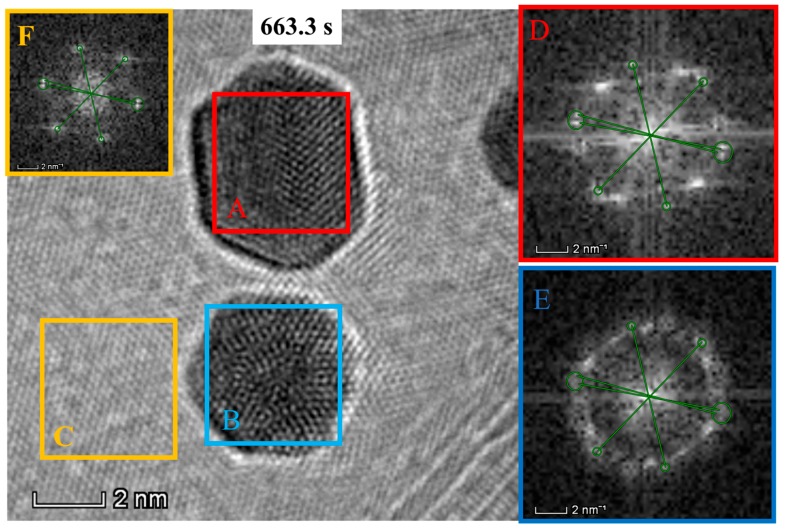
TEM images at the state of 0.1 s before the two Au NPs merging into one. The inset images (D, E and F) demonstrate the diffraction patterns by fast Fourier transformation (FFT) for the Au NPs and BNNT substrate. The diffraction patterns (inset images) corresponding to the images are highlighted by same colors (i.e., red, blue, and yellow colors). Diffraction patterns of hexagonal-symmetric BNNT were observed in all of the inset images that are noted by the green lines and circles.

To gain more insights into the particle mergence, the moment of last snap, i.e., 0.1 s before the two Au NPs merging, was recorded (note that this was actually extracted from the video because direct imaging is limited by the camera exposure speed) to investigate the detailed changes in particle states. The resulting images are shown in Figure 4, in which the diffraction patterns via fast Fourier transformation (FFT) of two Au NPs and BNNT substrate are also presented for the comparison purpose. Distinct six-fold diffraction patterns are associated with the substrate hexagonal symmetric BNNT, which were seen in all the images i.e., Figure 4D–F (highlighted by the green lines and circles). The Au nanoparticle “A” mainly maintains its lattice structure, but also exhibits surface atomic disorder with diffused and elongated diffraction points, which gives an unchanged zone axis of [110] orientation when comparing to the state with short-time irradiation (Figure 4A-inset). This indicates that the Au nanoparticle only occurs the in-plane rotation in this case that may be related to the relatively regular hexagonal shape with uniform charging force under the e-beam. While the sharp contrast is that the Au nanoparticles “B” nearly loses its atomic ordering, as well as the crystallinity and size, transforming into a group of randomly-arranged atoms (Figure 4B), which could be regarded as a type of plasma atomic gas with extremely high surface energy [23].

**Figure 5:**
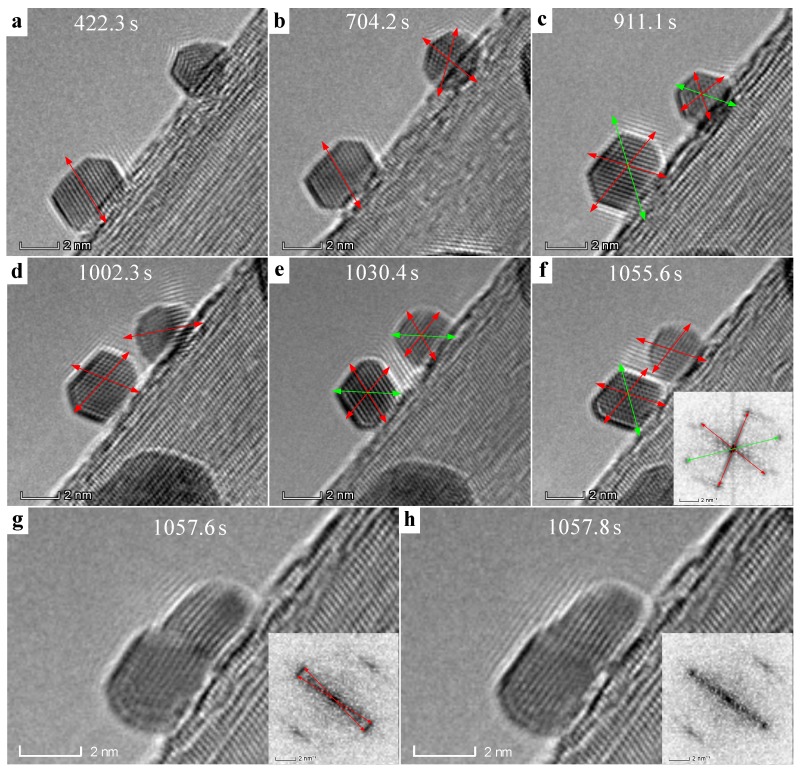
**Time series of TEM image extract from the video (Supplementary Materials-2).** Long-range motion and subsequent growth process of Au NPs on BNNTs at a lateral view under TEM e-beam irradiation in-situ recorded at different time. The Au NPs situated on the edge of BNNT underwent rotation, movement, shape change, and finally growth process. The orientation of the Au NPs were noted by the arrows: red and green denote [111] and [220] directions, respectively.

To acquire a comprehensive investigation for the rotation and mergence behaviors, in-situ TEM observation with a lateral view was performed. Without of questions, similar behaviors and phenomena were observed, as shown in Figure 5, in which two Au nanoparticles were also taken into consideration. Overall, they experienced a similar process of rotation, movement, and final mergence under the e-beam irradiation (detailed video was seen in Supplementary Materials-2). However, the orientations of these two Au nanoparticles changed with irradiation time (noted by the arrows: red and green denote [111] and [220] directions, respectively) and, further, the Au particles sometimes may have changes in shape, e.g., the images collected between 1002.3 s and 1055.6 s (Figure 5d–f). When the particles slip as a whole on the edge of BNNT, no lattice contact was observed between Au and BNNT, only indicating a physical absorption again without chemical bonding. These two Au particles finally merged into one particle in a very short period of less than 0.1 s, consistent with the results that were obtained from Figure 3. Careful observation reveals that the finally merged particle presents a feature of a single crystal (see the clear lattice finger in Figure 5h) without any parasitical interfaces, only followed by a quick adjustment in slight orientation discrepancy within 0.2 s (Figure 5g). Similar behaviors were also found in a three-particle system where the mergence was completed in a certain sequence (Supplementary Materials-3). 

As is clearly shown that the electron beams governed the particles’ movement and mergence above, how the intensity of the electron beams, as well as the substrate, contribute to such behaviors is necessary to be clarified. To this, similar experiments with lower beam current density (medium density ~5.0 × 10^4^ A/m^2^ and low density ~<1.54 × 10^4^ A/m^2^, respectively) were conducted. Hereof, as other experiment conditions remain unchanged, similar phenomenon was still observed for medium case (Supplementary Materials-5), while no obvious movement/rotation behaviors were observed at low current density (Supplementary Materials-3). Furthermore, for comparison purpose, the experiments for AuNPs@CNTs system under the same conditions were also carried out in order to evaluate the role that the substrate plays in (Supplementary Materials-4). It is well-known that the most important difference for CNTs and BNNTs is that the former is well conductive, while the latter is highly insulating. That is, the charging effect for CNTs under e-beams should be not dominant and is thus neglectable. This complies with the experimental findings that the Au particles on CNTs did not underdo significant rotation, movement and mergence, except for the slight displacement that is caused by the shrink of carbon nanotubes under e-beam irradiation [24]. This is somewhat similar to the reported motion and mergence phenomena for metal particles on CNTs that are mainly induced by the shrinkage and damage from substrate CNTs [25], obviously in a different mechanism with the present AuNPs@BNNTs. To conclude, the observed particle motion dynamic behaviors are distinct from previous studies.

## 4. Discussion

**Figure 6:**
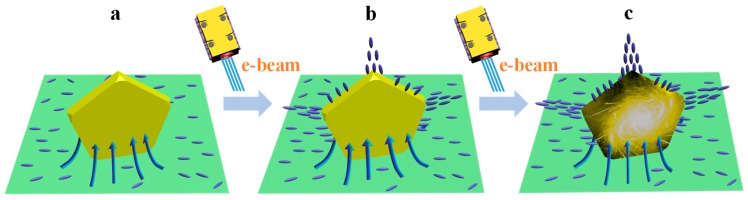
**Schematic diagram of mechanism.** Activated states evolution of Au nanocrystals under continuous e-beam irradiation. The blue olive shape denotes the charges. Arrows direct to the directions of the charge flows. Sharp area of the Au particles would accumulate more static charges according to electromagnetics theory that are also presented.

A metal particle with irregular shape in an electric field, as revealed by electromagnetics theory, would possess a certain charge distribution of like charges with more charges existing at the sharp surfaces. However, how could the two Au NPs carrying like charges move closer and finally merge into one particle? This seems not complying with the classical theory, but actually for real metal NPs system, structural changes at atomic level should be considered. To this, we proposed a new mechanism to illustrate the involved physical picture, as shown in Figure 6, which describes the activated states for an Au nanoparticle on BNNTs. Continuous e-beam irradiation would result in the charge accumulation (a charging phenomenon) on the BN surface. These charges would spontaneously flow from insulating BN to conductive Au, making Au nanoparticles overcharge (Figure 6a). Note that the AuPNs@BNNTs is distinct from the Metal@Carbon system. For the latter, due to both metal and carbon (e.g., CNTs or graphene) being highly conductive, there would not be too many charges transferring to the metals, while instead, carbon under long-term e-beam irradiation could incur deformation and shrinkage [24]. Such damage looks like a sort of motion behaviors under in-situ TEM. 

**Figure 7:**
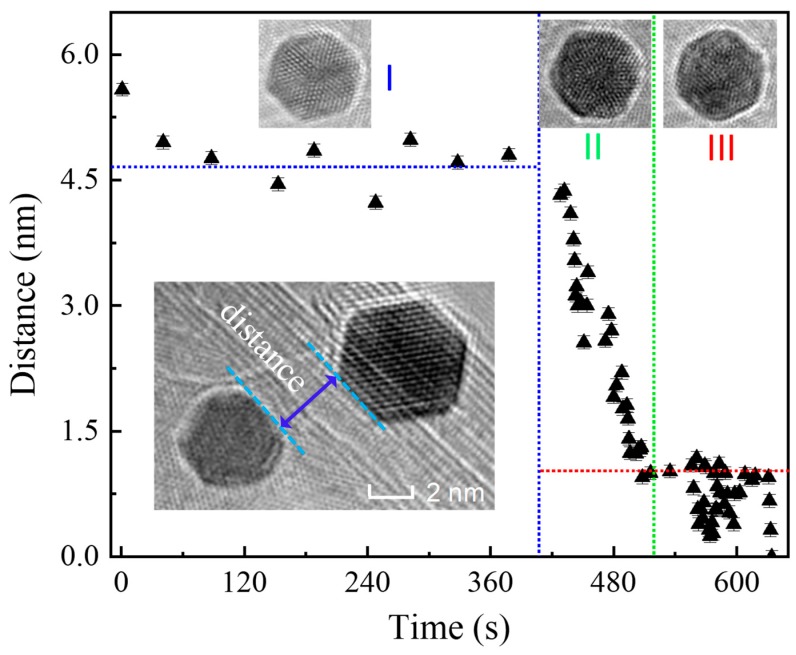
Distance between two Au NPs considered as a function of illumination time of the e-beam before final crystal growth. Three-section processes with distinct variation trends in distances were shown (separated by dash lines) to demonstrate different behaviors of Au NPs. The states of the Au NP in each section were also presented (inset I, II and III). The definition of the distance between two Au NPs under consideration was given (inset).

The motion dynamics of Au NPs, according to the varied trends in distances, experiences three correlated but different stages when considering their time-dependent distance for a two Au-NPs system. These three stages could be instantiated as: charging, activation, and adjacence, as seen in Figure 7 that presents the distance change between two Au NPs with a function of irradiation time before final growth. The charging stage lasted a quite long duration e.g., for 420 s. This process is mainly related to the charge transfer (Figure 6a), resulting in Au NPs being gradually subject to be heavily electrified. In this case, the Au NPs could sustain complete shape, crystallinity, and atomic ordering, thus only occurring the occasional rotation that is determined by the charging driving force (electric Coulomb force) [26,27]. This force is associated with the shape of the Au PNs as well as the state of the e-beam. Any fluctuations in charging driving force could promote the rotation motion or slight movement for the Au PNs, because Au PNs are not uniform in sizes and shape-irregular, and the e-beam is not exactly steady and centrical. As a consequence, such motion is dominated by the rule-less and non-directional rotation without apparent distance shortening. 

**Surface activation driven long-range movement.** The Au NPs started to move oppositely in a certain speed under the e-beam irradiation of after 420 s (Figure 7). This could not be interpreted, as we pointed out earlier, by a simply electromagnetic interaction, because the Au NPs thereof carrying like charges should move away from each other. Instead, the attraction should be related to the surface atomic disorder and diffusion i.e., surface activation, or in another word, surface ionization/plasma as the Au NPs are over charging with high-density static charges all the way upon e-beam (seen in Figure 4, Figure 5 and Figure 6c) [28]. The activated Au NPs are subject to a state of possessing extremely high surface energy, thus being highly instable to release energy through particles’ interactions and movements. The occurrence of such motion would overcome the resistance from the substrate BNNTs. The physisorption contact between Au NPs and BNNTs stems mainly from weak van de Waals interactions [29], with an energy of 0.05–0.5 eV [30]. Experiments also showed a smaller friction force of around sub-nN to several nN in metal/BN when comparing to that of metal/graphene interfaces. As a result, such a small energy and/or force would not impede the Au NPs motion with high energy. From the distance between two Au NPs under consideration, Au NPs move smoothly uniformly on BNNTs (Figure 7). The closer distance of two Au NPs accelerates the high charge density as well as the surface charging/activations. 

**Surface activation driven crystal growth.** The continuous surface activation promotes getting closer for Au NPs. When the distance of the Au NPs reduces down to a critical scale e.g., near 1 nm (after irradiation over 500 s, as seen in Figure 7), the movement becomes slowing down. That is, the distance was almost no longer shortened in a quite long duration from approx. 520 s to 620 s, until the last snap, the two NPs merge into one particle. The Au NPs in this state are over charging with high static charges that were localized at the surface to cause strong disorder and rather diffusive for the Au atoms, forming a sort of high-energy plasma [31,32]. Therefore, a single Au nanoparticle would experience not only an interaction sort between the surface-charge-density excitations and the electrons, but also the interactions between surface localized plasmons of the two particles that play a more important role in subsequent mergence, which is a consequence of the hybridization of the single-particle modes [33]. Strong interactions thus cause the apparent fluctuations and changes in the orientation, surface structure, and shapes of the Au NPs. 

**Figure 8:**
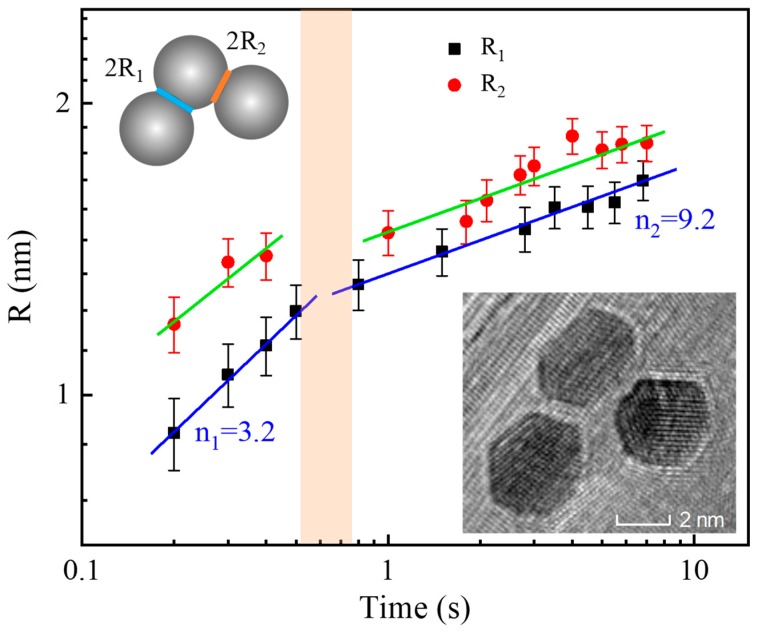
Neck areas (R) of the Au NPs in a three-particle system as a function of illumination time (t) of the e-beam, i.e., *R* vs. *t* (in logarithm scale). Both R_1_ and R_2_ follow two-section linear process (separated by stripe) as fitted (solid lines). To better demonstrate the neck area, a scheme of three-ball model is presented (inset: left-top) to illustrate the determination on the distance, which is performed on a real three-particles system (inset: right-bottom). The detailed growth process can be seen in Supplementary Materials-3.

As a result of the surface activation release when the Au NPs are within the state of critical distance, the mergence of two Au NPs into one happens to reduce the extremely high surface energy in order to reach a new stable state. The new formed particle presents good crystallinity, regular atomic ordering, and clear shape without any residual interfaces (Figure 5 and Supplementary Materials-3), which is obviously a crystal growth process. It can be attributed such crystal growth to Ostwald ripening other than the orientation attachment mechanism. The growth process was also monitored in-situ in a three-particle system to record the radius of the neck area (R: R_1_, and R_2_ are defined in Figure 8-inset) that is plotted as a function of time in Figure 8, which is normally subject to a typical growth process that is described by the following formula [34],

$$R={\left(\frac{Bt}{R_{i}^{m-1}}\right)}^{1/n}=Ct^{1/n}$$

where *t* is the growth time, *m* and *n* are determined by the diffusion mechanism, *C* is a growth-related constant that includes *R_i_* and *B* (*R_i_* is the initial radius of nanoparticles, and *B* is the growth constant, depending on the temperature and diffusion) [34]. It is worth noting that the growth for both *R*_1_ and *R*_2_ contains different two linear sections, as separated at a time scale of 0.6–0.7 s (highlighted stripe). Linear fitting on *R* vs. *t* (in logarithm scale) yields the n_1_ = 3.2 and n_2_ = 9.2, respectively. We noticed that both *R*_1_ and *R*_2_ have same trends with also the same n_1_ and n_2_, indicating the same growth processes and mechanisms. Doubtlessly, the present three-particle or dual-particle system does not follow the conventional thermal sintering steps [23,35]. Instead, n_1_ = 3.2, i.e., the short-time section, represents the process of lattice diffusion mechanism, while n_2_ = 9.2, i.e., the longer-time section, suggests a surface-dominated diffusion process. This is completely opposite to the previous Au/Ag nanoparticles system [36], but it is reasonable in our case, because, before growth, the Au NPs are in an activation state with greatly atomic disorder, diffusion, and extremely high surface energy in the Au lattice.

## 5. Conclusions

We performed an in-situ electron-beam continuous irradiation study on the Au nanocrystals that freely stand on the nitride boron nanotube to trace the structural evolution and the dynamic behaviors for real electrified metals. It was found, for the first time, that Au nanoparticles (2–4 nm) experienced anomalous long-range motion and subsequent crystal growth in surprisingly high rates, which is closely associated with the high density static charging induced surface activation. The growth process, opposite to previous observations, firstly undergoes the lattice diffusion and subsequently the surface-dominated diffusion mechanism. These abnormal phenomena that metals carrying like charges enabled inter-attraction and motion suggest structural changes and evolution should be concerned for the real metal systems that are under electron charging. This work thus highlights the importance and proposes new insights in understanding the dynamics behaviors for real metal nanocrystal systems that are helpful in the exploration design, synthesis, and structural-property correlations, etc.

## Supplementary Materials

The following are available online at https://www.mdpi.com/2079-4991/9/3/328/s1, Additional information on the evolution of temperature increase induced by the electron beam. TEM micrographs (Figure S1) and movies (Movies 1–5).
