# Supplementary material for: High Density Static Charges Governed Surface Activation for Long-Range Motion and Subsequent Growth of Au Nanocrystals
Source: Nanomaterials (Basel). 2019 Mar 1;9(3):328. doi: 10.3390/nano9030328 (PMC6473974; doi:10.3390/nano9030328)
Supplement: Supplementary file 1 [file nanomaterials-09-00328-s001.zip › nanomaterials-440578-supply.pdf]

Supporting Information for

High density static charges governed surface activation for long-range motion and subsequent growth of Au nanocrystals

Guoxin Chen,[1,4] Changjin Guo,[2] Yao Cheng [3], Huanming Lu [1], Junfeng Cui [1], Wanbiao Hu,[2,*] Rongrong Jiang [1]and Nan Jiang[1,*]

[1] Key Laboratory of Marine Materials and Related Technologies, Zhejiang Key Laboratory of Marine Materials and Protective Technologies, Ningbo Institute of Materials Technology & Engineering, Chinese Academy of Sciences, Ningbo 315201, P. R. China.

[2] School of Materials Science and Engineering, Yunnan University, Kunming 650091, P. R. China.

[3] Fujian Institute of Research on the Structure of Matter, Chinese Academy of Sciences,, Fuzhou,350002, P. R. China

[4] University of Chinese Academy of Sciences, 19 A Yuquan Rd., Shijingshan District, Beijing 100049, P. R. China.

Correspondence: Jiangnan@nimte.ac.cn (N. J.), huwanbiao@ynu.edu.cn (WB. H.).

This Supporting Information includes:

- Evolution of temperature increase induced by the Electron Beam
- Figures S1
- Captions for Movies S1 to S5

Other Supporting Information includes:

- Movies S1 to S4

Evolution of temperature increase induced by the Electron Beam

In order to estimate the temperature increase generated by TEM irradiations, the model proposed by V. G. Gryaznov[1] was applied. Thereof, the main consideration is that the homogeneous electron flux heats the NPs by the energy loos of the incident beam, and that the NPs only transfer heat through the contact zone with the substrate. The contact area between the NPs and the substrate is dependent on their radius. The mean temperature increase is given by:

$$\overline{T} = 1.06 \frac{a^2 J}{k_p e} \overline{\varepsilon_b}$$

where a is the radius of NPs, J the current density of the electron beam, k_p the thermal conductivity of the NPs, e the electron charge and $\overline{\varepsilon_b}$ the linear stopping power (energy loss per unit length) calculated by the Bethe theory.

The operation condition we used was a beam current density of 1.54×10^6 A/m^2 at 200 keV. The total stopping power for Au is 1.466 MeVcm^2/g for incident electrons of 200 keV. The radius of Au NPs used was 2.2 nm, and the thermal conductivity was calculated as[2]:

$$k_p = k_b \frac{1}{1 + \frac{\lambda e, b}{2a}}$$

where the sub index *b* is referred to bulk material, and $\lambda_{e,b}$ the mean free path of the electrons in bulk materials ($\lambda_{e,b}$ =36.14 nm). For Au NPs of 2.2 nm radius the thermal conductivity is ~28 W/m·K.

With these conditions, the temperature increase of the NPs induced by the electron beam can be negligible (lower than 1 K). Thus, the increase in temperature is not important in the movement of the NPs.

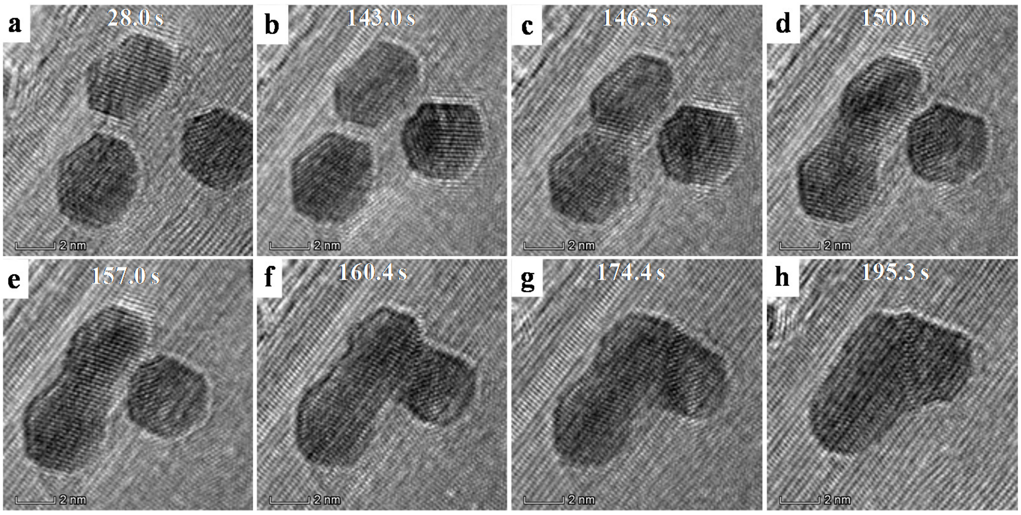

Figure S1. The structure evolution process of three Au NPs standing on the BNNT under electron beam irradiation.

Movie Captions:

Movie S1: The video was taken at the position where two Au nanoparticles standing on the BNNT substrate.

Movie S2: The video shows the rotation, movement and final mergence under the e-beam irradiation of two Au nanoparticles standing on the BNNT edge.

Movie S3. The behavior of Au NPs on BNNT under low beam current density (Video)

No obvious movement of the Au nanoparticles was observed when using relatively low the beam current density (<1.54×10^4A/m^2) to illuminate the samples.

Movie S4. The behavior of Au NPs on CNT under beam radiation (Video)

It is well-known that the most important difference for CNTs and BNNTs is that the former is well conductive while the latter is highly insulating. That is, the charging effect for CNTs under e-beams should be not dominant and thus neglectable. This complies with the experimental findings that the Au particles on CNTs did not underdo significant rotation, movement and mergence, except for the slight displacement caused by the shrinkage of carbon nanotubes under e-beam irradiation.

Movie S5. The behavior of Au NPs on BNNT under medium beam current density (Video)

Similar phenomenon compare to ~10^5A/m^2 was observed was observed when using medium beam current density (5×10^4A/m^2) to illuminate the samples.

Reference:

1 Gryaznov, V. G., Kaprelov, A. M. & Belov, A. Y. Real temperature of nanoparticles in electron microscope beams. *Philosophical Magazine Letters* **63**, 275-279, doi:10.1080/09500839108214655 (1991).
2 Warrier, P. & Teja, A. Effect of particle size on the thermal conductivity of nanofluids containing metallic nanoparticles. *Nanoscale Research Letters* **6**, 247, doi:10.1186/1556-276x-6-247 (2011).
